# The sociodemographic correlates of conspiracism

**DOI:** 10.1038/s41598-024-64098-1

**Published:** 2024-06-20

**Authors:** Adam Enders, Casey Klofstad, Amanda Diekman, Hugo Drochon, Joel Rogers de Waal, Shane Littrell, Kamal Premaratne, Daniel Verdear, Stefan Wuchty, Joseph Uscinski

**Affiliations:** 1 Department of Political Science, University of Louisville, Louisville, KY 40292 USA; 2 Department of Political Science, University of Miami, 1300 Campo Sano Blvd., Coral Gables, FL 33146 USA; 3 Department of Psychological and Brain Sciences, Indiana University, Bloomington, 47405 USA; 4 School of Politics and International Relations, University of Nottingham, Nottingham, NG7 2RD UK; 5 YouGov United Kingdom, Cambridge, EC1Y 8RT UK; 6 Munk School of Global Affairs and Public Policy, University of Toronto, Toronto, M5S 3K9 Canada; 7 Department of Electrical and Computer Engineering, University of Miami, Miami, 33146 USA; 8 Department of Computer Science, University of Miami, Miami, 33146 USA

**Keywords:** Conspiracy theory, Intersectionality, Gender, Race, Age, Income, Education, Psychology, Human behaviour

## Abstract

Despite hundreds of studies examining belief in conspiracy theories, it is still unclear who—*demographically*—is most likely to believe such theories. To remedy this knowledge gap, we examine survey data containing various operationalizations of conspiracism across diverse sociopolitical contexts. Study 1 employs a 2021 U.S. survey (n = 2021) to examine associations between sociodemographic characteristics and beliefs in 39 conspiracy theories. Study 2 similarly employs a survey of 20 countries (n = 26,416) and 11 conspiracy theory beliefs. Study 3 reports results from a 2020 U.S. survey (n = 2015) measuring perceptions about which groups are engaging in conspiracies. Study 4 interrogates data from nine U.S. surveys (2012–2022; n = 14,334) to examine the relationships between sociodemographic characteristics and generalized conspiracy thinking. Study 5 synchronizes studies 1–4 to provide an intersectional analysis of conspiracy theory belief. Across studies, we observe remarkably consistent patterns: education, income, age (older), and White identification are negatively related to conspiracism, while Black identification is positively related. We conclude by discussing why conspiracy theories may appeal most to historically marginalized groups and how our findings can inform efforts to mitigate the negative effects of conspiracy theories.

A growing body of work shows that beliefs in conspiracy theories are associated with various non-normative behaviors^1^, such as vaccine refusal^2^, criminality^3^, and political violence^4^. In response, social scientists have attempted to identify common factors associated with conspiracy theory beliefs^5^, finding that *sociodemographic traits* (i.e., age, racial identity, sex, education, income)^6–9^ frequently predict beliefs in conspiracy theories. Further, the growing literature provides rich theoretical support for the idea that the group identities^11^, group competition^12^, and group deprivation and mistreatment^13^ associated with sociodemographic traits are important ingredients of conspiracy theorizing.

That said, conspiracy theory research has proceeded in a piecemeal fashion, with most studies seeking to explain beliefs in only one or a few conspiracy theories at a time^14^. As a result, previously identified relationships between sociodemographic characteristics and conspiracy theory beliefs vary widely across the literature, depending largely on the specific conspiracy theory belief(s) researchers choose to investigate. Such an approach has resulted in a failure of the broader literature to clearly identify which empirical patterns are generalizable across conspiracy theories and which are situationally or contextually dependent^15^. For example, some studies find that men are more likely than women to believe conspiracy theories^8^, while others find that women are more likely^16^. Some studies find that older people are more likely to believe conspiracy theories^17^, whereas others find that younger people are more likely^17,18^. Some studies find that whites are more likely to believe in conspiracy theories^14^, although other studies conclude that racial minorities are more likely^19^. Findings are even inconsistent within particular groups; for example, Black Americans are more likely than other racial groups to believe that COVID-19 was spread on purpose, but less likely to believe that the threat of COVID-19 was exaggerated intentionally for political purposes^20^. These conflicting findings prevent the production of generalizable knowledge, leaving researchers and policymakers ill-equipped to effectively address conspiracy theory beliefs across social groups. Thus, the literature could benefit from additional studies examining intergroup differences to better understand the relative importance of various sociodemographic traits for conspiracy theorizing.

Although some scholars have attempted to better understand the sources of conspiracy theory beliefs amongst particular demographic groups, in many cases the research designs were capable of revealing only *intra*group, rather than *inter*group, differences^21^. Other studies focused on the effect of one demographic factor across a population (e.g., educational attainment), but could not adjudicate the *relative importance* of different demographic factors in predicting conspiracy theory beliefs^22^. Furthermore, the concept of intersectionality^23^ has yet to be integrated into the study of conspiracy theorizing, even though the theories and findings from the intersectionality literature speak directly to the group-centric factors that are hypothesized to motivate conspiracy theorizing. In this paper, we examine the relationships between sociodemographic characteristics and conspiracy theory beliefs using a large corpus of data with the intent of better understanding which sociodemographic characteristics are most predictive of conspiracy theorizing across space, time, and measures of conspiracism.

Given the urgent need to combat the non-normative behaviors associated with conspiracy theory beliefs^1^, we report results from cross-sectional data from 21 different countries and a time-series of U.S. surveys spanning a decade and employing multiple operationalizations of conspiracism. Study 1 reports results from a 2021 U.S. survey (n = 2021) examining the associations between sociodemographic characteristics and beliefs in 39 conspiracy theories. Study 2 uses survey data from 20 additional countries (n = 26,416) to compare associations between sociodemographic characteristics and beliefs in 11 conspiracy theories. Moving away from the idiosyncrasies inherent in specific conspiracy theories, Studies 3 and 4 investigate more general forms of conspiracism. Study 3 reports results from a 2020 U.S. survey data (n = 2015) investigating the relationships between sociodemographic characteristics and public perceptions of which groups and organizations are currently engaging in conspiracies. Study 4 reports time series data on the correlations between sociodemographic characteristics and conspiracy thinking style (i.e., the general predisposition to believe conspiracy theories) across nine U.S. surveys (2012–2022). Finally, Study 5 takes an intersectional approach to conspiracism—to our knowledge, the first of its kind—by considering combinations of sociodemographic characteristics in relation to conspiracism.

Across all studies, we observe remarkably consistent patterns. On the one hand, and in line with several previous studies^6,24^, education, income, age (older), and White identification tend to be negatively related to conspiracism; on the other, Black identification (in the U.S.) tends to be positively related, while both sex and Hispanic identification are weakly and inconsistently related. We conclude by discussing the value of our findings not only for scholars seeking to understand how conspiracy theory beliefs are adopted but also for practitioners and policymakers seeking to attenuate the negative consequences of conspiracy theory beliefs. We also consider how conspiracy theory beliefs held by members of marginalized groups may require interventions that go beyond those currently offered in the literature^25^.

## Expectations

Most people believe one or a few conspiracy theories. Indeed, the more conspiracy theories researchers ask about on a survey, the fewer the number of respondents who claim to believe in none of them^26^. This suggests that no specific demographic group has a monopoly on conspiracy theorizing. That said, the extant literature theorizes (i) why particular conspiracy theories might appeal more to some demographic groups than others, and (ii) why certain demographic groups might be more prone to engaging in conspiracy theories in general.

There is seemingly an infinite number of conspiracy theories that vary in terms of who they accuse, what they attempt to explain, and who the victims of the supposed plot are^27^. Numerous studies demonstrate that members of different social and political groups find some conspiracy theories more appealing, believable, or epistemologically warranted than others^15,28–30^. For example, people are attracted to conspiracy theories that accuse outgroups of conspiring and/or paint their ingroup as the victim; conversely, people are less attracted to conspiracy theories that accuse their ingroup of conspiring or that pertain to issues that their ingroup considers irrelevant^29,31–33^.

Moreover, socio-political context partially determines how prevalent conspiracism will be among various demographic groups^5^. How much power groups have in relation to each other^30,34^, how groups perceive and interact with each other^35,36^, and how much abuse, discrimination, and mistreatment groups have faced foster differential levels of conspiracism^13^. Groups who lack power and have been victimized by discrimination and abuse tend to be most prone to conspiracy theorizing, potentially since greater cognitive availability of historical trauma prompts individuals to view the world through a lens of conspiracy^37^. It is also common for individuals to perceive that they are victims of alleged conspiracies when their social group(s) have been victims of actual conspiracies^13,21,38–40^.

Here, we focus on age, racial and ethnic identity, sex, educational attainment, and income. While Studies 1–4 focus on each of these factors separately, as this is typically how they are operationalized in empirical studies, Study 5 takes an intersectional approach by examining these sociodemographic traits in tandem. Based on previous literature, we extrapolate several expectations regarding the relationships between demographics and conspiracism.

Regarding age, older people tend to be socially, politically, and economically engrained in society. Younger adults, on the other hand, tend to wield less social and political power, be less affluent than their older peers, and less established in their current communities and jobs (e.g., less property ownership, fewer children). Moreover, studies of adolescents show that beliefs in conspiracy theories take root early on^41^. Taken together, we expect younger adults to be more likely to see sinister forces working against them in society relative to older adults.

Educational attainment could make beliefs in conspiracy theories less likely by fostering a reservoir of knowledge^42^, critical thinking skills^43^, and scientific ways of thinking^22^. Education could also stimulate career and finance-related opportunities that make individuals materially better off, which could also, in turn, lead to fewer conspiracy theory beliefs^44^. As such, we expect higher levels of educational attainment to be associated with lower levels of conspiracy theorizing. We note as well, that education could make some people more likely to believe conspiracy theories^9^; for example, the critical thinking skills afforded by education could lead to or buttress conspiracy theory beliefs^43,45^.

Higher levels of income could, by virtue of providing security, material comfort, and some measure of success, make conspiracy theories seem less likely because ‘winners’ have little to complain about; conversely, those facing economic deprivation often look for explanations for their deprivation in conspiracy theories^46^. It may be the case that a lack of material wealth leads to a lack of feeling of control over one’s circumstances^47^. As such, we expect income to be negatively related to conspiracism.

Some demographic characteristics are tied to social identities^48^, which foment group competition and animate all manner of group dynamics^35,36,49^. For example, the literature is rich with accounts of racial, ethnic, and sex-based groups accusing outgroups of conspiring against them^14,49–53^. Past and present discrimination, maltreatment, and subjugation, including victimization by actual conspiracies, may also foster conspiracism by providing historical evidence of outgroup malignance. Given their historical mistreatment, we expect that Black Americans^13^ are especially suspicious of the intentions of other groups, while White Americans may be more resistant to such suspicions given their privileged status. This logic may also apply to other minority groups, such as Hispanic Americans, though our expectations are less crystalized as the previous literature is thinner.

Given that women have traditionally been excluded from positions of sociopolitical power^54^, possessed fewer and less evenly applied rights, and been victims of patriarchal practices^55^, women may be more likely to expect mistreatment^56^ and, therefore, believe in more conspiracy theories than men. On the other hand, men respond differently to potential threats, potentially prompting them to be more prone to conspiracism than women^8^. Men are also more likely to engage in violence and exhibit higher levels of dark triad traits^57^, both of which are strong correlates of many conspiracy theory beliefs^58^. Given the discord in existing literature, we do not derive a clear expectation about differences in conspiracism by sex.

As for Study 5, we provide the first intersectional approach to the study of conspiracism. As many of the limited number of non-intersectional analyses from previous work are at odds with each other, we do not have clear expectations regarding the results our intersectional analysis. However, we emphasize the importance of taking this first step toward recognizing the potential significance of intersectional characteristics and identities with respect to conspiracism. Just as psychological and political traits may combine into constellations of dispositions that foster or dampen conspiracism, we expect that sociodemographic characteristics may exhibit similar patterns, significantly bolstering our understanding of who conspiracy theorists are.

## Materials and methods

This research was performed in accordance with all relevant guidelines and regulations, and with the Declaration of Helsinki. All survey respondents provided informed consent by checking a box on a computer screen and could leave each survey at any time. Institutional Review Board approvals, where applicable, were obtained from University of Miami (Date, Protocol Number: 9/24/2012, 20120757; 9/13/2016, 20120757 (MOD00013692); 7/07/2018, 20120757 (MOD00023764); 7/07/2019, 20190623; 3/15/2020, 202000095; 6/03/2020, 20200673; 10/07/2020, 20201154; 3/15/2021, 20210244; 5/13/2022, 20220472).

## Study 1

An important question involves the generalizability of previous findings across individual conspiracy theories. To better understand the relationships between beliefs in specific conspiracy theories and sociodemographic traits, we begin by examining beliefs in a large number of conspiracy theories that vary in numerous ways. This analysis will reveal how (or if) the relationships between conspiracy theory beliefs and sociodemographic traits vary (or generalize) from conspiracy theory to conspiracy theory.

### Data

We fielded a survey on 2,021 U.S. adults from April 30 to May 19, 2021, in partnership with Qualtrics. The sample, which utilized an opt-in, quota-based design, is reflective of the U.S. adult population in terms of age, sex, educational attainment, race and ethnicity, and household income based on 2019 American Community Survey estimates. Additional details about the human subjects approval (IRB approval 20210244), response quality protocols, and the sociodemographic composition of the sample appear in the Supplemental Information (SI).

#### Dependent variables

Participants expressed their agreement with 39 conspiracy theories. Question wording and the percentage of respondents who endorsed each conspiracy theory appear in Table SI 4. We note that all of the conspiracy theories have been polled previously, either by scholars or by mainstream polling houses (e.g., Gallup, Roper)^59^, and all adhere to the following definition of “conspiracy theory”^27^: an idea in which a small group of powerful people are working in secret and for their own benefit, against the common good, and in a way that undermines the bedrock ground rules against the use of force and fraud^27^. The definition we employ also has an epistemological facet: conspiracy theories are ideas that have yet to be demonstrated to be likely to be true by experts possessing appropriate domain-relevant knowledge and who employ data and methods that can be openly verified and challenged by others^60^. That said, we note that epistemologists and other scholars have presented a multitude of definitions of “conspiracy theory” featuring different epistemological standards for what does and does not count^61–64^.

**Figure 1:**
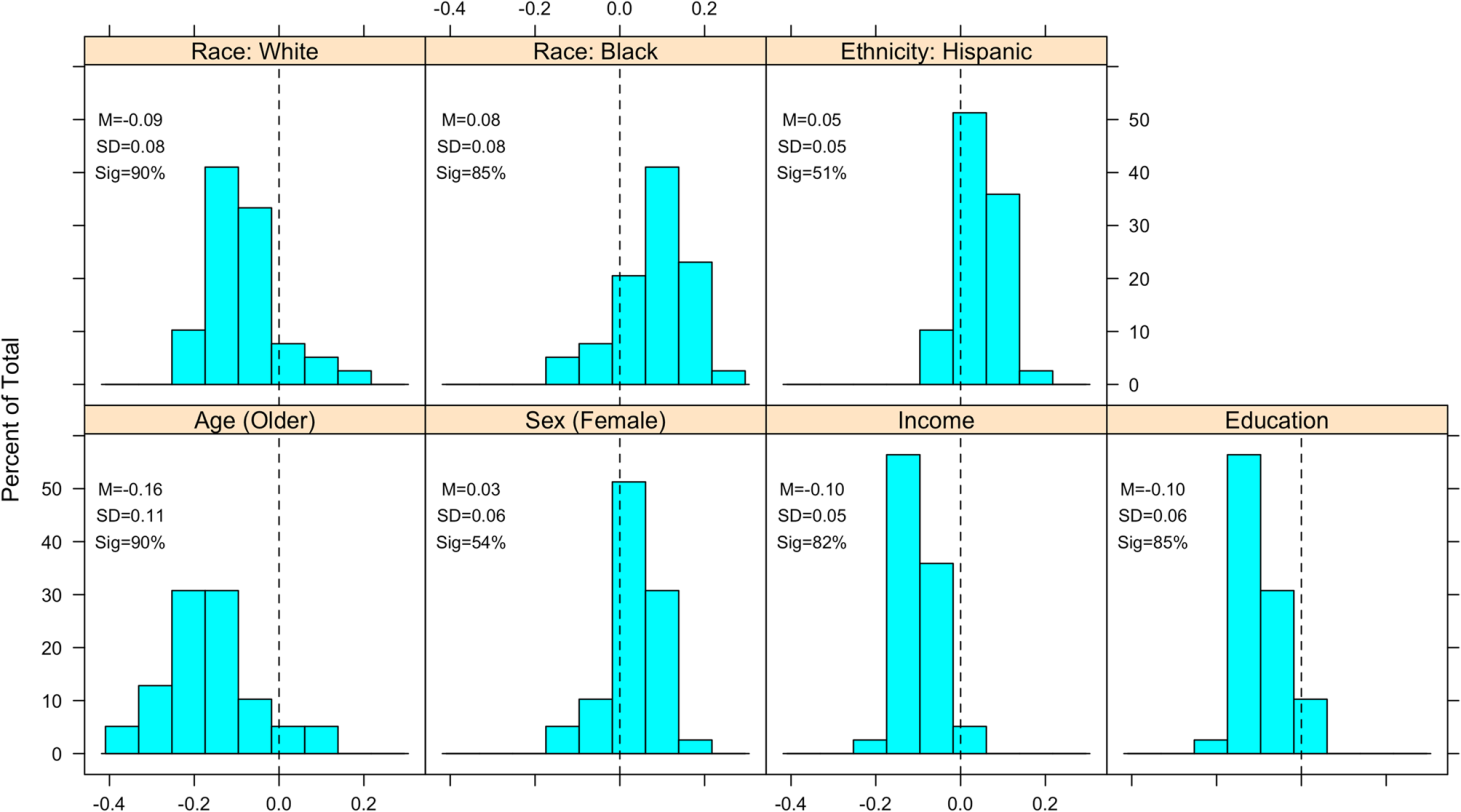
Distribution of correlation coefficients, by sociodemographic characteristics, across all conspiracy theory beliefs. Mean, standard deviation, and percentage of cases where correlation was statistically significant (*p* < 0.05) appear in each panel. *P*-values corrected for multiple comparisons via the Benjamini–Hochberg procedure.

**Figure 2:**
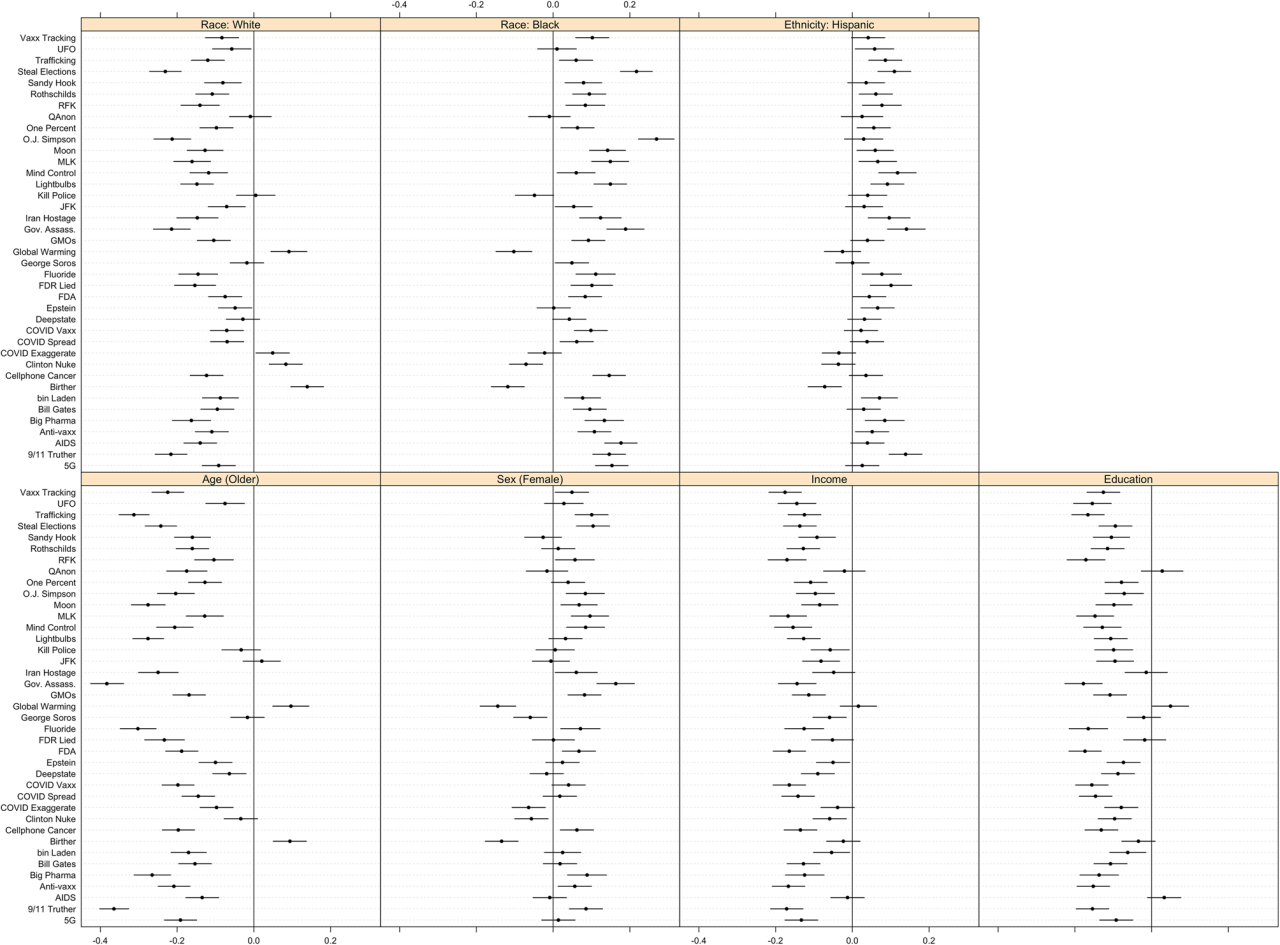
Correlation between each conspiracy theory belief and each sociodemographic characteristic. Horizontal bars are 95% confidence intervals.

Many conspiracy theories attract different sets of believers^58^ and there is no “correct” set of conspiracy theories yet devised to speak to the whole of conspiracy theorizing; however, available evidence suggests choosing a large number of conspiracy theories that involve various topics is an appropriate strategy^58,65^. Thus, we polled a larger number of conspiracy theories than are usually employed by social scientific studies (39 items), and we intentionally chose a wide variety of theories varying by topic (e.g., government, medicine, technology, aliens), salience (e.g., COVID-19 versus the moon landing), and alleged conspirators (e.g., politicians, scientists, the wealthy). Between 56% (JFK assassination) and 5% (Osama bin Laden is alive) of respondents believed the theories we asked about. We show the results of all the conspiracy theories aggregated (Fig. 1) and disaggregated by individual conspiracy theory (Fig. 2). In addition, we later turn to more general measures of conspiracism that eschew the idiosyncrasies of individual conspiracy theories and focus on (i) perceptions that particular groups are conspiring in some way^59^ and (ii) the generalized predisposition towards conspiracy theorizing^66^.

#### Independent variables

We examine age (greater values indicate greater age), sex (positive values indicate identification as female), race and ethnicity, education (greater values indicate greater levels of formal education), and household income (greater values indicate higher income). For race and ethnicity, we restrict our analysis to White, Black, and/or Hispanic identifiers due to the sample sizes of smaller racial and ethnic groups. Table SI 5 contains the measurement characteristics and distribution of these variables.

### Methods

We computed the correlation coefficient between each conspiracy theory belief and each sociodemographic, resulting in 273 unique correlations. We present the distribution of correlation coefficients across conspiracy theory beliefs, in addition to the average correlation, standard deviation, and the proportion of statistically significant correlations at *p* < 0.05 (using Benjamini–Hochberg to correct for multiple testing). In the SI, we also report distributions of coefficients from multivariate models that include all these sociodemographic characteristics (with Benjamini–Hochberg corrected *p*-values).

### Results

Figure 1 displays the distribution of correlations between each sociodemographic characteristic and each conspiracy theory belief. The weakest correlate is sex (female), which is statistically significant in only 56% of cases (22/39 beliefs) with an average correlation of 0.03 and a standard deviation of 0.06 (tied with education for second smallest). Compared to White and Black identification, Hispanic identification exhibits the weakest average correlation of the three at 0.05 and is only significant in 54% of cases. We observe more negative relationships for White identifiers (85% are negative, 90% are significant at *p* < 0.05) and more positive relationships for Black identifiers respondents (85% positive, 85% are significant at *p* < 0.05), though the size of these relationships are typically small (average correlations of − 0.09 for White identification and 0.08 for Black identification). Correlations with educational attainment and household income both have an average correlation of -0.10 with standard deviations between 0.05–0.06, and are statistically significant in 82–85% of cases. Finally, age exhibits the strongest average correlation at − 0.16 and is statistically significant in 90% of cases, suggesting that older individuals tend to be less likely to believe conspiracy theories than younger individuals. These relationships are consistent with the expectations outlined above.

In the SI, we display coefficients from regression models of each conspiracy theory belief on each sociodemographic characteristic, controlling for each of the other characteristics. As expected, we find fewer cases across all variables where estimates are statistically significant as sociodemographic characteristics are correlated with each other. With controls, we find the most consistently significant effects (in the same average direction) for age and income, with fewer significant effects for educational attainment and sex. When it comes to race and ethnicity, White and Hispanic are never statistically significant, while self-identification as Black is statistically significant about 50% of the time. The difference between the bivariate correlations and the regression results is likely the consequence of an inherently high level of multicollinearity between sociodemographic characteristics.

Figure 2 displays each of the 273 individual correlations used to construct the distributions in Fig. 1. Although it is easier to make inferences from the distributions, there is at least one noteworthy pattern in Fig. 2. In contrast to most conspiracy theory beliefs, the Global Warming and Birther conspiracy theories are positively associated with White identification and age (older), and negatively associated with Black identification, Hispanic identification (in the case of Birther only), and sex (i.e., more self-identified males believe these than females). Notably, these findings align with previous studies of these conspiracy theories^67,68^, and suggest that scholars should not attempt to draw generalizable conclusions from just one or a few conspiracy theories (in particular these two conspiracy theories) given that they attract different audiences.

In total, our analysis of the relationships between 39 conspiracy theory beliefs and sociodemographic traits reveals how these relationships vary across conspiracy theories. Older and White respondents tend to agree with fewer conspiracy theories in our survey while Black respondents and those with less educational attainment and lower incomes tend to agree with a greater number of conspiracy theories. As Fig. 1 suggests, while some demographic traits appear to have generalizable relationships across conspiracy theories, the relationships do vary, particularly in the case of some conspiracy theories (e.g., those about Obama’s birth certificate and global warming).

## Study 2

To better understand whether the relationships observed in Study 1 apply to countries outside of the U.S., Study 2 employs survey data from 20 additional countries in order to examine the associations between sociodemographic characteristics and beliefs in conspiracy theories.

### Data

**Figure 3:**
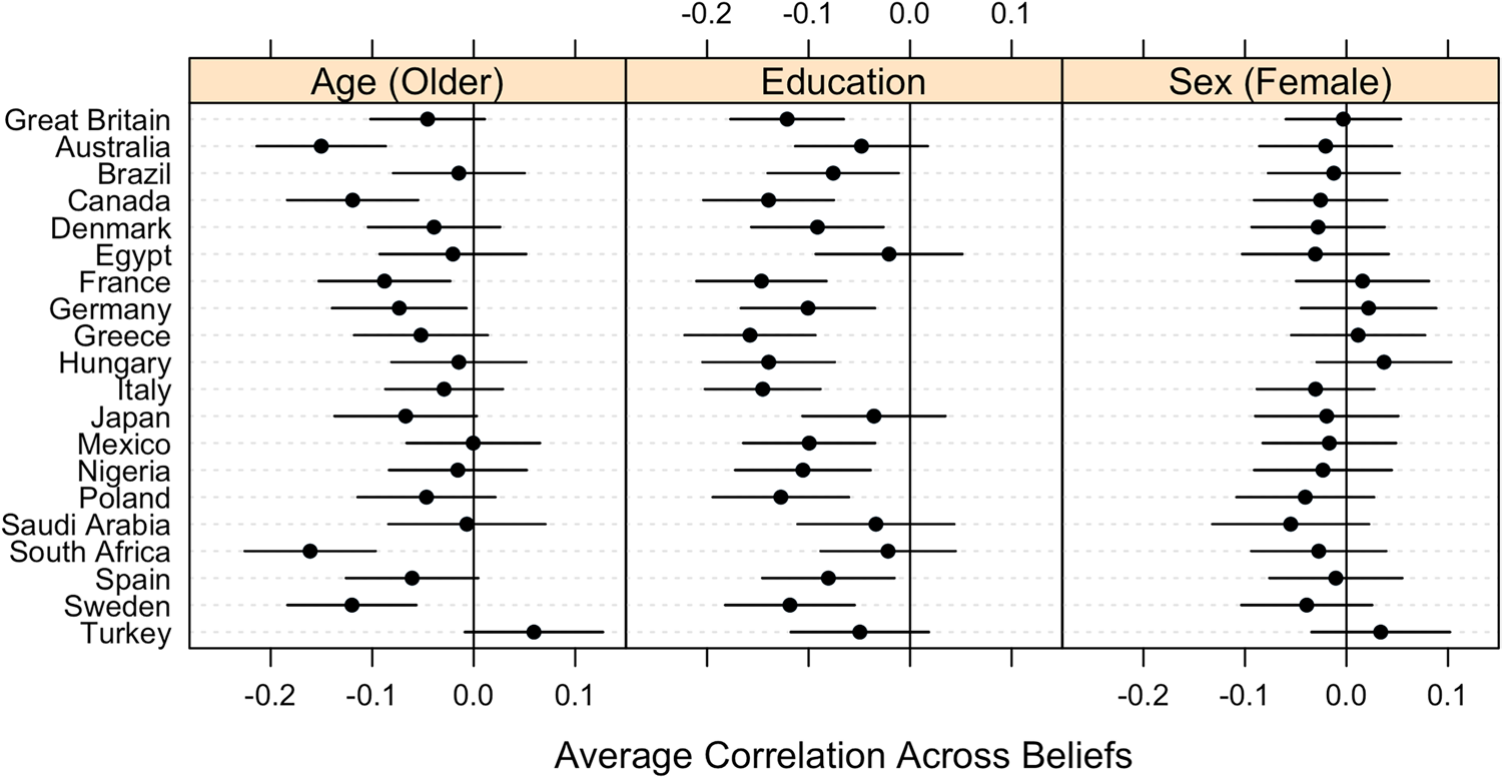
Average correlations between specific conspiracy theory beliefs and sociodemographic characteristics, by country. Horizontal bars represent 95% confidence intervals.

We employed surveys asking about 11 conspiracy theory beliefs across 20 countries spanning six continents (total n = 26,416) (see Fig. 3 for the full list). All surveys were conducted between July 30–August 24, 2020 by YouGov. YouGov constructs and maintains panels of individuals from which a final sample––that is representative of each country’s population––is constructed. Questions were approved and translated by YouGov and their partners in each country. The sociodemographic composition of each sample appears in the SI. This article represents independent use of YouGov data and does not necessarily reflect the analysis or interpretation of YouGov.

#### Dependent variables

We asked respondents about 11 different conspiracy theories ranging from COVID-19 and AIDS to the 9/11 attacks and the Holocaust, ubiquitous topics that transcend cultural barriers (see Fig. SI 3 for the average level of belief in each conspiracy theory for each country). Each conspiracy belief question, which appear in the SI, was answered using four-point scales ranging from “definitely” not true (1) to “definitely” true (4), with options of “probably” not true (2) and “probably” true (3) in the middle.

#### Independent variables

As race/ethnicity and household income were excluded from this data collection, we focus on age, sex, and educational attainment. See the SI for the composition of each country-sample based on age, sex, and educational attainment.

### Results

Figure 3 displays the correlations between conspiracy theory beliefs and age, education, and sex. We find that the correlations with age and educational attainment are either significantly negative (i.e., older individuals are less likely than younger individuals to believe conspiracy theories) or insignificant in every instance. While such results comport with both our expectations and our findings from Study 1, we also observe that sex is never significantly correlated.

In the SI, we disaggregate these data by conspiracy theory, presenting the 219 individual correlations across countries (20 countries * 11 conspiracy theories, minus 1 for the missing Holocaust denial item in Germany) for each sociodemographic characteristic. The average correlation across all country/conspiracy theory pairs (presented in Figs. SI 4–SI 6) is − 0.05 for age (Range = − 0.30–0.14, *SD* = 0.08; i.e., older individuals are less likely to believe conspiracy theories), − 0.09 for educational attainment (Range = − 0.23–0.08, *SD* = 0.06; i.e., individuals with more formal education are less likely to believe conspiracy theories), and − 0.01 for sex (Range = − 0.17–0.14, *SD* = 0.06; i.e., males are very slightly more likely than females to believe conspiracy theories). The correlations with age are significant at *p* < 0.05 in 50% of the 219 cases while the significance rate is 64% for educational attainment and 29% for sex.

In sum, our findings demonstrate that age and educational attainment tend to negatively correlate with conspiracy theory beliefs; these findings are congruent with those from Study 1. Thus, we provide evidence that *some* relationships between sociodemographic characteristics and beliefs in conspiracy theories hold across geographic boundaries.

## Study 3

Studies 1 and 2 focused on beliefs in specific conspiracy theories. Study 3 seeks to examine the relationships between sociodemographic characteristics and broader forms of conspiracism that circumvent the idiosyncrasies of specific conspiracy theories. Here, we focus on the social and political groups that individuals believe are likely to be working in secret “against the rest of us.”

### Data

We fielded a survey on 2,015 U.S. adults from October 8–21, 2020 in partnership with Qualtrics. The sample, which utilized an opt-in, quota-based design, is reflective of the U.S. adult population based on age, sex, educational attainment, race and ethnicity, and household income according to 2019 American Community Survey estimates (see SI for details about the sociodemographic composition of the sample).

#### Dependent variable

**Figure 4:**
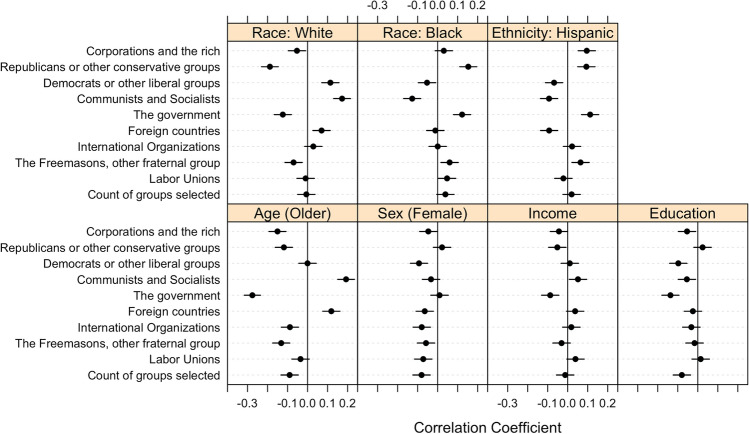
Correlation between selecting each group as a likely conspirator and sociodemographic characteristics. Horizontal bars represent 95% confidence intervals.

Respondents were asked the following question: “Which of these groups are likely to work in secret against the rest of us? Please check all that apply.” Respondents were able to select from nine pre-determined groups, which appear in Fig. 4, or select “none of the above.” We also generated a count of the 9 groups each respondent selected, which ranges from 0–9.

#### Independent variables

We consider the same sociodemographic characteristics as in Study 1, including age, sex, race and ethnicity, educational attainment, and household income. All demographic characteristics are measured as described in Table SI 5.

### Results

Figure 4 shows the correlations between each sociodemographic characteristic across each potential conspirator. In particular, positive correlations suggest that individuals that belong to the sociodemographic group or possess a greater degree of the sociodemographic characteristic (e.g., income) are more likely to perceive a given group as “likely to work in secret against the rest of us,” and vice versa. On average, the correlations across the groups of potential conspirators in Fig. 4 tend to mimic the average correlations from Fig. 1 in that the correlations with age (greater values indicate older individuals), sex (positive values indicate identification as female ), educational attainment, and White identification tend to be negative, while correlations with Black and Hispanic identification tend to be positive. However, we find that the correlations with income are evenly split, directionally, across conspirators.

Figure 4 also shows that, while the results are inconsistent across potential conspirators, White respondents, in line with our expectations, select slightly fewer conspirators (significant correlations with 3/9 groups) than do Black (4/9 significant correlations) and Hispanic (4/9 significant correlations) identifiers. Looking more closely at these relationships, Whites are more likely to perceive Democrats, communists, socialists, and foreign governments as likely conspirators. This observation may be explained by the fact that White individuals (compared to other racial/ethnic groups) tend to align themselves more with the Republican Party and conservative ideologies^69^, which are typically more hawkish on foreign affairs and emphasize anti-communism. Perhaps as a consequence, Whites are more likely believe conspiracy theories accusing the left, communists, and foreign entities^46^. Black and Hispanic identifiers are more likely to perceive corporations and the rich, Republicans and other conservative groups, the government, and Freemasons and other fraternal groups as conspirators. Likewise, Black and Hispanic individuals are less likely to perceive Democrats, other liberal groups, communists, and socialists as conspirators. We also find that Hispanics are less likely than non-Hispanics to perceive foreign countries as likely conspirators. These patterns align with the partisan and ideological tendencies of Blacks and Hispanics (i.e., more liberal, Democratic)^69^, which are putatively based on a history of subjugation by powerful political and social entities (e.g., the government, corporations)^21^, and, in the case of Hispanics, a recent history of immigration from other countries.

As for age, the patterns in Fig. 4 support our expectations and mimic the findings of Study 1 and 2, with younger individuals being more likely to select more of the groups as conspirators. However, older individuals are more likely to choose “communists and socialists” and “foreign countries” as conspirators, which is potentially rooted in the fact that older Americans grew up during the Cold War and were socialized to fear communism and foreign influence. We tend to observe substantively weak and statistically non-significant correlations across conspirators when it comes to sex, educational attainment, and income. For all three characteristics, the only statistically significant correlations are negative. For education and income, our findings comport with the observations from Studies 1 and 2.

In short, the correlations presented in Fig. 4 tend to mimic the average correlations from Fig. 1. But, just as with Fig. 1, the correlations vary across measures of conspiracism, in this case, across which group of conspirators we ask respondents about.

## Study 4

Like Study 3, Study 4 examines a more general form of conspiracism—the general predisposition to see events and circumstances as the outcomes of conspiracies, *conspiracy thinking*^15^. Since this measure has been repeatedly polled on (in the U.S. context) for more than a decade, we also present the correlations between sociodemographic characteristics and conspiracy thinking over the course of a decade to understand how such relationships change (or not) over time.

### Data

We utilize data from nine nationally representative surveys of U.S. adults fielded between October 2012 and June 2022. Each of the samples, which vary in size between 1,000 and 2,023, were fielded by either Qualtrics or the Cooperative Congressional Election Study/YouGov. All samples are broadly representative of the adult population based on age, sex, race, and educational attainment, although we note that the sampling procedure is quota-based. We present the sociodemographic composition of each sample, as well as additional details about the size of each sample, when they were fielded, and how samples were constructed, in the SI.

#### Dependent variable

We measured conspiracy thinking, defined as the predisposition to interpret events and circumstances as the product of conspiracies, using the American Conspiracy Thinking Scale (ACTS)^46^. This previously validated measure^70^ is correlated with a wide range of beliefs in specific conspiracy theories^15^ and stable over time^59^. The ACTS (Range = 1–5, *M* = 3.10–3.35, *SD* = 0.83–1.04, α = 0.76–0.87 across surveys) is an index of responses to the following four questions, which are measured using five-point, “strongly disagree” (1) to “strongly agree” (5) scales:

1. Much of our lives are being controlled by plots hatched in secret places.
2. Even though we live in a democracy, a few people will always run things anyway.
3. The people who really “run” the country are not known to the voters.
4. Big events like wars, the current recession, and the outcomes of elections are controlled by small groups of people who are working in secret against the rest of us.

#### Independent variables

We examine the same sociodemographic characteristics as in Study 1, including age, sex, race and ethnicity, educational attainment, and household income. In seven of our nine surveys, all demographic characteristics are measured exactly as they were in Table SI 5, above (the sociodemographic composition of each sample is available in the SI). For our first three surveys, fielded in October 2012, 2016, and 2018, the only difference is that income is a finer-grained measure.

### Results

**Figure 5:**
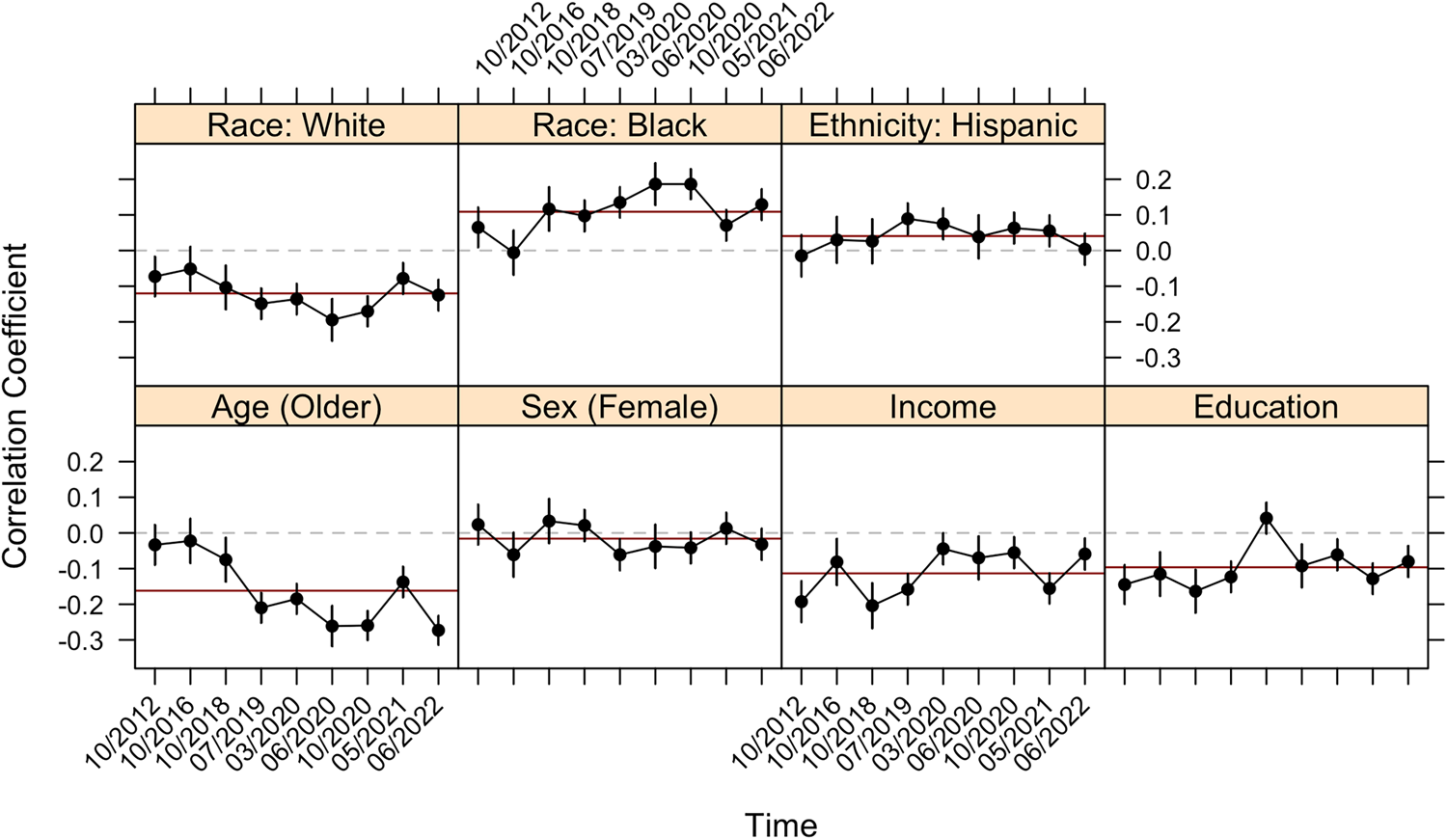
Correlation between the ACTS and sociodemographic characteristics over time, with 95% confidence intervals. Horizontal red line represents the average correlation across all surveys.

The correlations between each sociodemographic characteristic and the ACTS, by year/survey, are presented in Fig. 5. While we do not find any obvious, systematic trends in the relationships over time, age shows a weak, albeit statistically significant, linear trend ($\beta =-0.028$, *p* = 0.011), indicating that older individuals tend to exhibit lower levels of conspiracy thinking than younger individuals. This trend is largely driven by the weaker negative association between conspiracy thinking and age in 2012 and 2016. This finding could be rooted in the fact that these two surveys were administered during the presidency of Barack Obama, who was popular with young people^71^. Considering the relationships with White and Black identification in these two years, we find that White identification has a more positive relationship compared to the rest of the series, while Black identification has a slightly stronger negative relationship with conspiracy thinking. We surmise that this is also owed to Obama, who was strongly supported by Black Americans (hence less conspiracism) and less supported by Whites^72^.

The correlations with education, age, household income, and White identification are generally negative, while those with Black identification are generally positive. The correlations with Hispanic identification and sex (positive values indicate identification as female) are generally non-significant. The average correlations, across years, are: − 0.16 for age (significant in 7/9), − 0.10 for education (significant in 8/9), − 0.02 for sex (significant in 1/9), − 0.11 for income (significant in 9/9), 0.11 for Black identifiers (significant in 8/9), 0.04 for Hispanic identifiers (significant in 4/9), and − 0.12 for White identifiers (significant in 8/9). In every case, all statistically significant correlations are in the same direction as the average correlation across time.

The average correlations in Fig. 5 (horizontal red lines) are remarkably similar to the average correlations between sociodemographic characteristics and the 39 different conspiracy theory beliefs in Fig. 1. Indeed, the average difference (i.e., across all sociodemographic characteristics) between the average correlations (i.e., those presented in Study 1 and Study 3) is only 0.02, with a range of 0 (i.e., no difference at all; this applies to age and education) to 0.05 (sex). This pattern showcases how the conspiracy thinking predisposition behaves, empirically, like an average of beliefs in many specific conspiracy theories––precisely the pattern we would theoretically expect from a predisposition, which is an upstream cause of specific beliefs^58,73^. If researchers are interested in understanding the causes, consequences, and general correlates of the general tendency toward conspiratorial thinking, a measure of the predisposition––the ACTS in this case––appears to be an accurate and economical method that avoids the inferential pitfalls associated with specific beliefs showcased in previous studies.

## Study 5

Finally, in Study 5 we build from Studies 1 and 4 by combining sociodemographic traits together to investigate how groups of sociodemogaphic traits––rather than individual traits––might jointly encourage conspiracism. We utilize data from previous studies to conduct this intersectional analysis^23^, employing both beliefs in specific conspiracy theories and conspiracy thinking.

### Data

For the first analysis (using conspiracy theory beliefs), we utilize the May 2021 U.S. survey employed in Study 1, which contains beliefs in 39 specific conspiracy theories. For the latter analysis (using conspiracy thinking, as measured with the ACTS), we combine all 9 U.S. datasets employed in Study 4, resulting in an aggregated dataset of 14,334 respondents. This aggregated dataset is useful for our purposes, as intersectional analyses typically suffer from low sample sizes within combinations of characteristics, especially when those characteristics represent statistical minorities. While we still cannot examine more complicated three-way combinations of characteristics due to sample size limitations, we can examine two-way combinations with a minimal sample size of 312 respondents, which is sufficient for making robust statistical inferences.

### Results

**Figure 6:**
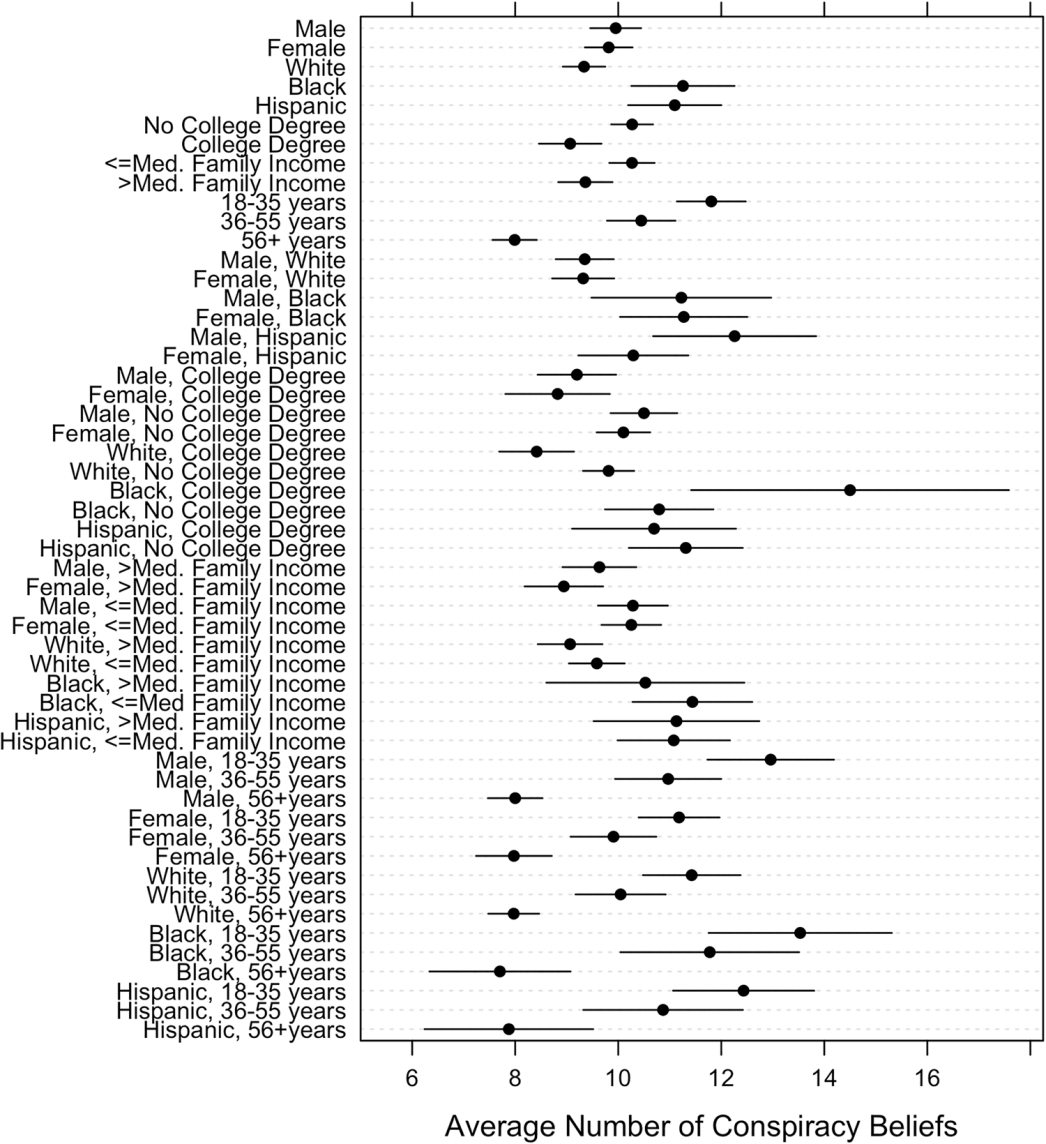
Correlations between a count of 39 conspiracy theory beliefs (see Study 1) and combinations of sociodemographic characteristics, with 95% confidence intervals.

In Fig. 6, we plot the average number of beliefs (out of 39 total) for various combinations of sociodemographic characteristics. Responses of “(strongly) agree” and “believe/yes,” depending on the question format (see Table SI 4), are considered to be expressions of beliefs. As the patterns from previous studies suggest, men and women do not differ much in the number of conspiracy theories they believe, while Black and Hispanic individuals report more beliefs than Whites. Furthermore, we find that respondents with a college degree report fewer beliefs than individuals with less education, while respondents below 2021 U.S. median family income ($71,000) reported more beliefs than their counterparts above the median and younger individuals reported more beliefs than older individuals. These patterns tend to hold across combinations of characteristics. For example, at the bottom of Fig. 6 we observe that the average number of beliefs among young people differs by race/ethnicity according to the pattern described above. We can also see that the age relationship is slightly stronger than the race/ethnicity relationships, though these are the two strongest correlations; hence, there is a difference of about 6 beliefs between young Black individuals and old White individuals.

We note two differences between patterns observed in Fig. 6 and expectations we might had given the results presented in Fig. 1. First, we observe a significant interaction between sex and Hispanic identification that we do not observe among Black and White individuals: Hispanic men believe more conspiracy theories than Hispanic women (diff = 1.97 beliefs, *p* = 0.035). We also find that Black individuals with a college degree hold more conspiracy theory beliefs than Black individuals who do not possess a college degree (diff = 3.71 beliefs, *p* = 0.016), contrary to the patterns we reported earlier which showed a fairly consistent negative correlation between education and conspiracism. Future work should keep in mind that race/ethnicity may interact with sex and education to promote conspiracy theory beliefs in unique ways that do not hold across other subgroups in the population.

**Figure 7:**
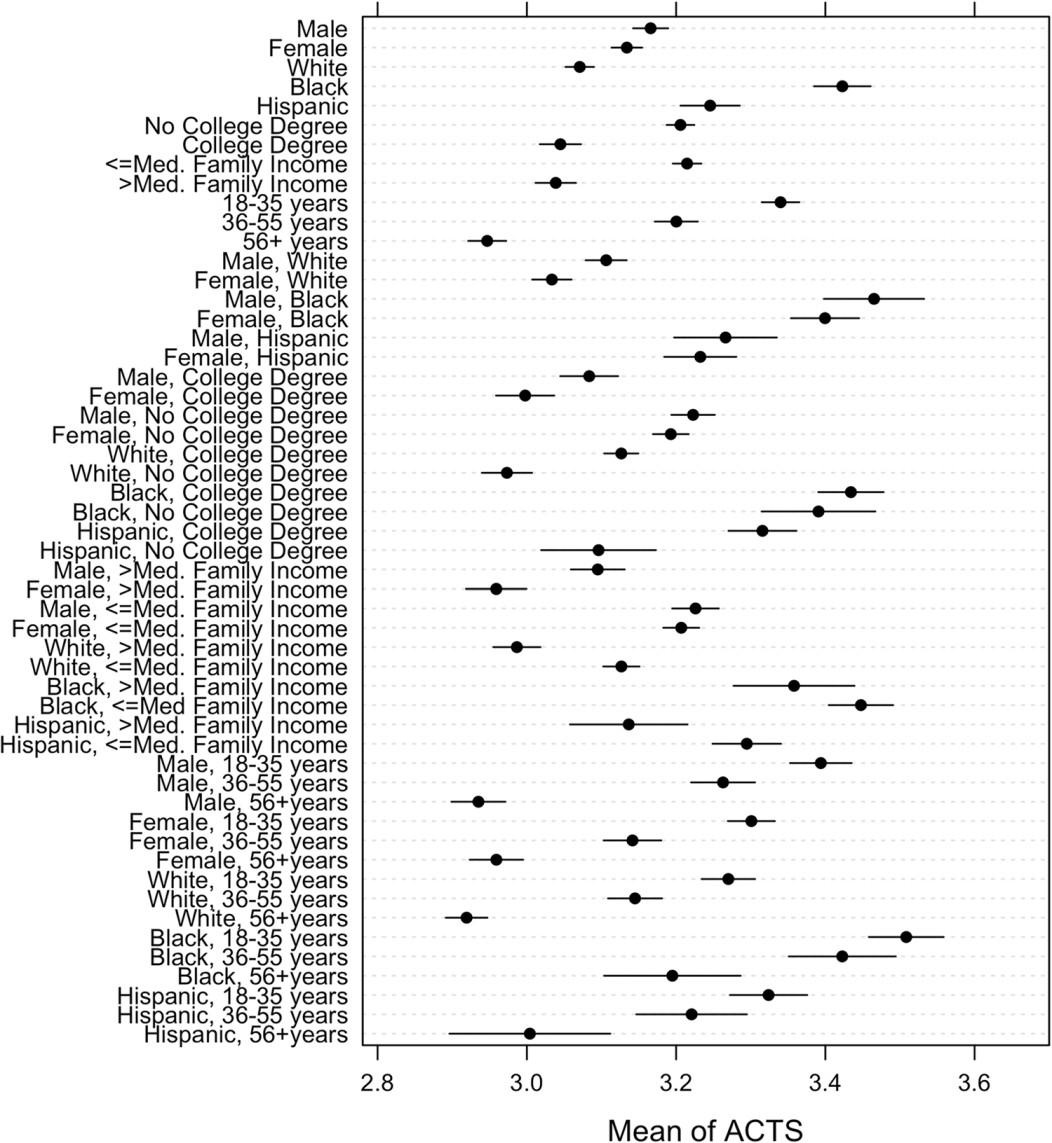
Correlations between the ACTS and combinations of sociodemographic characteristics, with 95% confidence intervals.

Next, Fig. 7 presents the average level of the ACTS by combination of sociodemographic characteristics. The patterns in Fig. 7 are remarkably similar to those in Fig. 6, as we should expect if the ACTS is a robust proxy for beliefs in specific conspiracy theories. We also observe similar peculiarities as in Fig. 6.

Although we do not observe a difference in conspiracy thinking between Hispanic men and women, we find a significant, albeit small, sex difference among Whites (diff = 0.07, *p* < 0.001; i.e., male showing higher levels than females). We also find that Whites with a college degree register higher levels of the ACTS than Whites without a college degree (diff = 0.15, *p* < 0.001). A similar pattern emerges among Black individuals, though the difference is not statistically significant (*p* = 0.322). Still, these patterns across operationalizations of conspiracism suggest that an intersectional approach to understanding conspiracy thinking is valuable and warranted. Simply put, it appears that sex interacts with race and ethnicity, and race/ethnicity with educational attainment, in unique ways that are not apparent from the simpler approach of examining one characteristic at a time.

### Ethical approval

Survey respondents provided informed consent via checking a box on a computer screen and could leave the survey at any time. This research was performed in accordance with all relevant guidelines and regulations, and with the Declaration of Helsinki. The Supplemental Information includes Institutional Review Board information where applicable.

## Discussion

While researchers have learned much about conspiracy theorizing, the relationship between conspiracy theory beliefs and sociodemographic traits remain contested in the literature, leaving scholars and practitioners unsure which social groups, if any, are most prone to conspiracism. By pinpointing who––sociodemographically––is likely to believe in conspiracy theories, researchers may be better positioned to address conspiracy theory beliefs and their negative effects.

Across five studies that included data from 21 different countries, numerous distinct operationalizations of conspiracy theory belief, and 11 years, we observed remarkably systematic patterns. First, income (U.S. samples only), educational attainment, and age tended to be negatively correlated with specific conspiracy theory beliefs and conspiracy thinking, whereas sex and Hispanic identification (U.S. samples only) tended to be weakly correlated and were statistically significantly correlated in only approximately 50% of cases. In the U.S. samples (Studies 1, 3, and 4), we also found that White identification tended to be negatively, albeit weakly, correlated with conspiracy theory beliefs and conspiracy thinking, while Black identification tended to be positively correlated.

While people who believe specific conspiracy theories tend to be younger, less educated, and less wealthy than those who do not believe in conspiracy theories, these relationships are dependent on the specific conspiracy theory beliefs in question and far from deterministic. Even though the patterns we observed are quite consistent, they also suggest that we cannot reliably identify conspiracy theorists by sociodemographic characteristics alone. Although explanations of conspiracism that focus on socioeconomic depravation or socioeconomic “loser” status may provide the bedrock upon which conspiracy theory beliefs can flourish, other traits and conditions such as psychological characteristics, political status and partisan identification, and a hospitable information environment may be necessary to bring conspiracism into full bloom.

The relationships involving sex, race, and ethnicity are, on balance, even more dependent on the specific conspiracy theory in question, with sex and Hispanic identification being significantly related only slightly more than 50% of the time across specific conspiracy theory beliefs. Moreover, even this very weak relationship between conspiracy theory beliefs and identification with racial and ethnic minority groups may be a natural reaction to past subjugation by powerful groups, some of which was conspiratorial in nature ^13,74^. Altogether, our findings suggest that researchers should more carefully and completely integrate sociodemographic characteristics, including combinations of such, into theories of conspiracism.

Our findings also provide a cautionary tale for researchers. We found that the Climate Hoax and Birther conspiracy theory beliefs behave differently than the other 37 conspiracy theories we examined in Study 1. The outlier status of these two conspiracy theories is noteworthy as they have been two of the most popular conspiracy theories for researchers to study for more than a decade^14,51,75–77^. Questions about these theories have even been included on large, publicly available surveys such as the *American National Election Study*, facilitating their examination to a greater degree than other conspiracy theories. This has likely led scientific inference and generalization down the wrong path in some instances since these two conspiracy theories systematically attract different groups of believers than do many––if not most––others. In short, researchers need to be cautious when attempting to make generalizable claims about conspiracism based on only one or a few conspiracy theories.

We end our investigation with a puzzle. Scholars have long noted how conspiracy theorizing may be a consequence of relative powerlessness or depravation^46^, both of which may be natural reactions to lived experiences. This is certainly the case with respect to individuals whose social groups have endured subjugation and even the perpetration of real conspiracies. In other words, patterns in the sociodemographic correlates of conspiracism may be interpreted to paint a sympathetic picture of at least some conspiracy theory believers. However, the same cannot be said of the psychological and political correlates of conspiracy beliefs. Narcissism, sadism, psychopathy, delusional thinking, support for political violence, and “broken” epistemologies––all consistent, stable correlates of conspiracy theory beliefs^10,58^––are widely considered normatively undesirable. At the very least, scholars need to account for the possibility that people are attracted to conspiracy theories because of structural circumstances that are out of their control and further consider whether conspiracy theory beliefs are always best thought of as pathological, a product of undesirable psychological and political tendencies. Understanding the institutional and structural roots of generalized conspiracy thinking could provide novel insights into the root causes of conspiracy theory beliefs and, in turn, insight into how they might be changed or mitigated.

Numerous authors have previously argued that at least some conspiracy theories are epistemically justified, in that the arguments and evidence in favor of such theories may warrant either further investigation or even belief^78–80^. While our findings do not speak to the truth quality of conspiracy theories, they do seem to provide support for argument that other researchers have expressed before: that––*at least sometimes*––beliefs in conspiracy theories are sociologically, politically, or psychologically justified, in that historical (or even present) marginalization by social and political institutions may make conspiracy theories attractive explanations for various phenomena^81–83^. Such a conclusion has serious, tangible implications for the development of strategies for preventing or “correcting” conspiracy theory beliefs^84^. Much of the extant literature is premised on the ideas that (i) conspiracy theories are inherently dubious, and that (ii) the people who believe in conspiracy theories suffer from cognitive limitations or personality defects^5,10^. However, if people believe in conspiracy theories because of the very real experiences of their social group—providing tangible evidence of the nefarious intentions of others that can be used to make reasonable inferences about the present or future—interventions based upon the aforementioned premises may not only be ineffective, but potentially unethical. The attempt to convince groups who have been subjected to documented abuse, discrimination, and real conspiracies that conspiracy theories are inherently false, or that their past experiences serve as a weak basis from which to make inferences about the future, would amount to epistemological gaslighting. In such a scenario, not only are interventions unlikely to prove efficacious, they may also backfire by sowing further distrust in experts and knowledge-producing institutions. Ultimately, certain manifestations of conspiracy theories may be less of a problem in need of a cure than a symptom of long-term mistreatment which has yet to be rectified. In this light, our findings pose serious theoretical questions to the rapidly growing industry of experts who are focused on reducing the spread of conspiracy theory beliefs.

### Limitations and future directions

Our cross-national study (Study 2) did not account for race and income; as such, we were not able to replicate our analyses involving race and income outside of the U.S. There are also some characteristics we did not examine, such as marital status and family size. We encourage future studies to expand our investigation, not only into more sociodemographic correlates, but more conspiracy theory beliefs and additional countries where racial and gender dynamics may differ from those in the U.S. Further, while we posited some of the mechanisms by which sociodemographic groups may become attracted to conspiracy theories (e.g., historical mistreatment), our analyses do not directly investigate the role of such potential mechanisms due to data limitations. We recommend that future studies build off our work by theorizing about and empirically exploring the potential reasons why members of particular sociodemographic groups may be more or less prone to conspiracy theorizing. Some researchers have already undertaken this important work^8,22,42^, yet basic questions remain unsettled. For example, the mechanisms connecting sociodemographic traits to conspiracy theory beliefs might function differently across countries, time, and context; therefore, more multi-country examinations are necessary. We believe that the generalizable patterns uncovered in the present manuscript can help guide future work in this vein. We also emphasize that sociodemographic characteristics may interact with situational influences, psychological traits, or political orientations. Future work might explore interactions between sociodemographic characteristics and other explanatory factors.

Our findings regarding age––that, in general, younger people are more conspiracy-minded—suggest that people do not, as is popularly theorized, become more conspiracy-minded over time through incidental exposure from politicians, fake news, and social media, but rather become less conspiracy-minded over time. That said, we cannot test this interpretation without longitudinal/panel data, which could be used to track individuals over time as they age^85,86^. A small number of recent studies employing panel data show that conspiracism is stable or declines over time^86,87^. Regardless, panel data could be used to answer many questions about the (primary) direction of causation between conspiracism and numerous other factors.

Finally, we encourage scholars to develop frameworks that can help guide choices regarding which conspiracy theories to study. For example, there may be conspiracy theory beliefs that are more strongly associated with violence or prejudice than others, and therefore more useful in some research contexts than others. With guidance such as this, researchers may be able to refocus conspiracy theory research on preventing harm in more efficacious ways.

### Supplementary Information

Supplementary Information.

## Data Availability

All data and materials required for replication are available on the Open Science Framework (OSF) https://osf.io/7wbs6/.
