# Supplementary material for: The sociodemographic correlates of conspiracism
Source: Sci Rep. 2024 Jun 20;14:14184. doi: 10.1038/s41598-024-64098-1 (PMC11190281; doi:10.1038/s41598-024-64098-1)
Supplement: Supplementary file 1 — Supplementary Information. [file 41598_2024_64098_MOESM1_ESM.pdf]

Supplemental Information:
The Sociodemographic Correlates of Conspiracism

Author List: Adam Enders[a], Casey Klofstad[b], Amanda Diekman[c], Hugo Drochon[d], Joel Rogers de Waal[e], Shane Littrell[f], Kamal Premaratne[g], Daniel Verdear[h], Stefan Wuchty[h], Joseph Uscinski[b]*

[a]Dept. of Political Science, Univ. of Louisville, Louisville, KY 40292, USA
[b]Dept. of Political Science, Univ. of Miami, Coral Gables, FL 33146, USA
[c]Dept. of Psychological and Brain Sciences, Indiana University, USA
[d]School of Politics and International Relations, Univ. of Nottingham, NG7 2RD, UK
[e]YouGov United Kingdom, EC1Y 8RT, UK
[f]Munk School of Global Affairs and Public Policy, Univ. of Toronto, M5S 3K9, Canada
[g]Dept. of Electrical and Computer Engineering, University of Miami, USA
[h]Dept. of Computer Science, University of Miami, USA

***Corresponding Author**: Dept. of Political Science, 1300 Campo Sano Blvd., Univ. of Miami, Coral Gables, FL 33146, USA; uscinski@miami.edu, (305) 746-2651

Table of Contents

I. Sociodemographic composition of sample for Study 1, page 2

II. Sociodemographic composition of sample for Study 2, page 3

III. Sociodemographic composition of sample for Study 3, page 4

IV. Question wording and variable measurement for Study 1, pages 5–7

V. Regression models for Study 1, pages 8–9

VI. Question wording for Study 2, page 10

VII. Average belief by country for Study 2, page 11

VIII. Complete correlations for Study 2, pages 12–14

IX. Sociodemographic composition of samples for Study 4, pages 15–17

I. Sociodemographic composition of sample for Study 1

Table SI 1: Sociodemographic information about original U.S. sample, compared to 2010 U.S. Census estimates.

Characteristic	2010 Census Estimate	Qualtrics May 2021
Age	38	48
High school degree	88	97
Some college or more	59	71
Female	51	50
White	72	62
Black	13	14
Hispanic	16	16
n		2,021

Note: All entries are percentages except age, which is the median.

While median age is slightly higher for our surveys than the 2010 Census estimates, recall that we are able to poll on only those age 18 years old and older, whereas the Census provides the median age of all Americans.

The University of Miami institutional review board approved this survey on April 8, 2021 (20210244). Respondents were provided an informed consent preamble before the survey began and could exit the survey at any time. We took two primary steps to ensure data quality. First, we excluded respondents who "sped" through the survey very quickly, operationalized as those who spent less time that one standard deviation below the median completion time upon a soft launch of the survey. Second, we excluded respondents who failed to pass four attention checks embedded in the survey—two standalone questions and two embedded in grids, per best practices[1].

II. Sociodemographic composition of sample for Study 2

All surveys were conducted between July 30–August 24, 2020 using an online interview administered to members of panels that YouGov constructed and maintain. Emails are sent to panelists selected at random from the base sample. The survey was written in English and translated into other languages by YouGov offices in those countries or certified partners. All YouGov studies are conducted in line with YouGov's Group Code of Conduct and Ethics and industry best practice. YouGov also has internal processes for secondary review of sensitive research topics. Race was not included as a standard demographic variable owing to cross-country inconsistencies of compliance, categorisation, and sample coverage. For all countries, quotas were applied on at least age, sex, and region. This article represents independent use of YouGov data and does not reflect the analysis or interpretation of YouGov.

Table SI 2: Sociodemographic information about Study 2 samples.

Characteristic	Australia	Brazil	Canada	Denmark	Egypt	France	Germany	Great Britain	Greece	Hungary
Female	51	51	53	52	36	58	52	53	50	54
18–24	9	16	8	9	34	7	9	12	9	10
25–34	18	22	11	21	39	13	13	12	14	15
35–44	20	22	15	13	13	15	15	17	17	19
45–54	16	18	17	16	9	18	15	18	19	18
55+	37	22	48	42	5	48	48	42	41	38
Upper secondary	15	32	25	18	13	48	24	17	26	29
Bachelors or equiv.	31	35	22	9	56	9	10	24	37	16
n	1,048	1,010	1,021	1,024	1,016	1,058	1,026	1,383	1,011	1,023

Characteristic	Italy	Japan	Mexico	Nigeria	Poland	Saudi Arabia	South Africa	Spain	Sweden	Turkey
Female	57	51	53	36	58	36	49	50	56	48
18–24	7	7	15	20	13	18	18	9	6	16
25–34	17	12	24	50	19	27	28	15	14	22
35–44	19	15	20	23	17	29	22	22	18	23
45–54	20	17	17	5	11	19	14	22	19	16
55+	37	50	23	1	40	8	18	32	43	23
Upper secondary	41	27	16	6	36	13	29	24	47	26
Bachelors or equiv.	7	46	44	65	8	52	33	27	13	43
n	1,337	1,018	1,025	1,007	1,001	1,003	1,001	1,022	1,047	1,007

Note: All entries are percentages. Education categories are % for which that is highest level; upper secondary is the equivalent of high school.

III. Sociodemographic composition of sample for Study 3

Table SI 3: Sociodemographic information about October 2020 sample, compared to 2010 U.S. Census estimates.

Characteristic	October 2020	2010 Census Estimate
Age (median)	43	38
High school degree (%)	97	88
Some college or more (%)	76	59
Female (%)	51	51
Household income (median)	$25,000–$49,999	$49,445
Race:		
White (%)	68	72
Black (%)	14	13
Hispanic (%)	17	16
n	2,015	

While median age is slightly higher for our surveys than the 2010 Census estimates, recall that we are able to poll on only those age 18 years old and older, whereas the Census provides the median age of all Americans.

The University of Miami institutional review board approved this survey on October 7, 2020 (Protocol #20201154). Respondents were provided an informed consent preamble before the survey began and could exit the survey at any time. To ensure data quality, we first excluded respondents who "sped" through the survey very quickly, operationalized as those who spent less time that one standard deviation below the median completion time upon a soft launch of the survey. Second, we excluded respondents who failed to pass four attention checks embedded in the survey—two standalone questions and two embedded in grids, per best practices [1].

IV. Question wording and variable measurement for Study 1

Table SI 4: Percent of participants who believe in each of 39 conspiracy theories.

Question wording	Percent Believe
1. Do you think one man was responsible for the assassination of President Kennedy, or do you think there were others involved?	56
2. The one percent (1%) of the richest people in the U.S. control the government and the economy for their own benefit.	52
3. Do you think the government is keeping information from the public that shows U.F.O.'s (Unidentified Flying Objects) are real or that aliens have visited the Earth?	50
4. Jeffrey Epstein, the billionaire accused of running an elite sex trafficking ring, was murdered to cover-up the activities of his criminal network.	48
5. There is a “deep state” embedded in the government that operates in secret and without oversight.	44
6. Do you feel that the Assassination of Senator Robert Kennedy was the act of one individual or part of a larger conspiracy?	43
7. The dangers of genetically-modified foods are being hidden from the public.	40
8. The number of deaths related to the coronavirus has been exaggerated.	36
9. The Food and Drug Administration is deliberately preventing the public from getting natural cures for cancer and other diseases because of pressure from drug companies.	35
10. Elites, from government and Hollywood, are engaged in a massive child sex trafficking racket.	34
11. Do you feel that the Assassination of Martin Luther King was the act of one individual or part of a larger conspiracy?	33
12. Hillary Clinton conspired to provide Russia with access to nuclear materials.	29
13. The dangers of vaccines are being hidden by the medical establishment.	29
14. Coronavirus was purposely created and released by powerful people as part of a conspiracy.	29
15. Billionaire George Soros is behind a hidden plot to destabilize the American government, take control of the media, and put the world under his control.	26
16. A powerful family, the Rothschilds, through their wealth, controls governments, wars, and many countries' economies.	26
17. The coronavirus is being used to force a dangerous and unnecessary vaccine on Americans.	24
18. Do you think the U.S. government has engaged in the assassination of entertainers who have tried to spread a counterculture message they didn’t like, such as John Lennon, Kurt Cobain, Tupac Shakur, and others, or not?	20
19. Do you believe that the pharmaceutical industry is in league with the medical industry to "invent" new diseases in order to make money, or not?	20
20. Health officials know that cell phones cause cancer but are doing nothing to stop it because large corporations won't let them.	20
21. Certain U.S. government officials planned the attacks of September 11, 2001, because they wanted the United States to go to war in the Middle East.	19

22. Barack Obama faked his citizenship to become president.	19
23. Some people have argued that President Franklin D. Roosevelt knew about Japanese plans to bomb Pearl Harbor but did nothing about it because he wanted an excuse to involve the U.S. (United States) on the side of the allies in the war.	19
24. Do you believe media or the government adds secret mind-controlling technology to television broadcast signals, or not?	18
25. Do you believe global warming is a hoax, or not?	18
26. Some people are hiding the truth about the December 14, 2012 school shooting at Sandy Hook Elementary in order to advance a political agenda.	16
27. Do you think there is, or is not, a national conspiracy to kill police?	16
28. Republicans won the presidential elections in 2016, 2004, and 2000 by stealing them.	15
29. Do you completely agree, mostly agree, mostly disagree, or completely disagree that AIDS is a form of systematic destruction of minorities like blacks and Hispanics?	15
30. Do you believe the government adds fluoride to our water supply, not for dental health reasons, but for other, more sinister reasons, or not?	13
31. The U.S. government is mandating the switch to compact fluorescent light bulbs because such lights make people more obedient and easier to control.	12
32. The coronavirus is being used to install tracking devices inside our bodies.	12
33. Do you think that the Reagan campaign made a deal with the Iranians to hold the American hostages in Iran until after the 1980 presidential election or not?	12
34. Bill Gates is behind the coronavirus pandemic.	11
35. Do you think there was a police conspiracy to frame O.J. Simpson or not?	10
36. Thinking about space exploration, do you think the government staged and faked the Apollo moon landings, or don't you feel that way?	10
37. 5G cell phone technology is responsible for the spread of the coronavirus.	7
38. Are you a believer in QANON?	6
39. Do you believe that Osama bin Laden is dead, or do you think he is still alive?	5

Note: Where response options are not dichotomous (e.g., yes/no, believe/don't believe), the proportion expressing belief is those who "agree" or "strongly agree" (rather than "strongly disagree," "disagree" or "neither agree, nor disagree") with a sentiment.

Table SI 5: Details about sociodemographic characteristics.

Variable	Range/Coding	Percent	Mean	Std. Dev.
Age	18–94		47.85	18.19
Sex	0–Male	49.98	0.50	0.50
	1–Female	50.02		
Education	1–No high school	3.02	3.56	1.46
	2–High school degree/GED	25.88		
	3–Some college, no degree	28.90		
	4–two-year college degree	10		
	5–four-year college degree	18.75		
	6–Advanced degree (MA, PhD)	13.46		
White	0–Not White	38.40	0.61	0.49
	1–White	61.60		
Black	0–Not Black	86.49	0.14	0.34
	1–Black	13.51		
Hispanic	0–Not Hispanic	83.67	0.16	0.37
	1–Hispanic	16.33		
Household Income	1–$24,999 or less	19.84	3.27	1.76
	2–$25,000–49,999	20.39		
	3–$50,000–74,999	17.32		
	4–$75,000–99,999	13.51		
	5–$100,000–149,999	18.01		
	6–$150,000–199,999	6.33		
	7–$200,000 or more	4.60		

V. Regression models for Study 1

Below, we reproduce Figure 1, subsetting by general question format. In Figure A1, we present the distributions of OLS regression coefficients for each predictor across conspiracy belief questions that employ ordinal response formats (e.g., five-point "strongly disagree" to "strongly agree"). In Figure A2, we present the distributions of logistic regression coefficients for each predictor across conspiracy belief questions that employ dichotomous response formats (e.g., agree vs. disagree, believe vs. do not believe). Even though these do not perfectly match each other in terms of average correlation, standard deviation, and proportion significant, the patterns are similar.

Figure SI 1: Distribution of OLS coefficients for ordinal conspiracy belief questions, by sociodemographic characteristic. Mean, standard deviation, and percentage of cases where coefficient was statistically significant (*p*<0.05) appears in text. *P*-values corrected for multiple comparisons via Benjamini-Hochberg procedure.

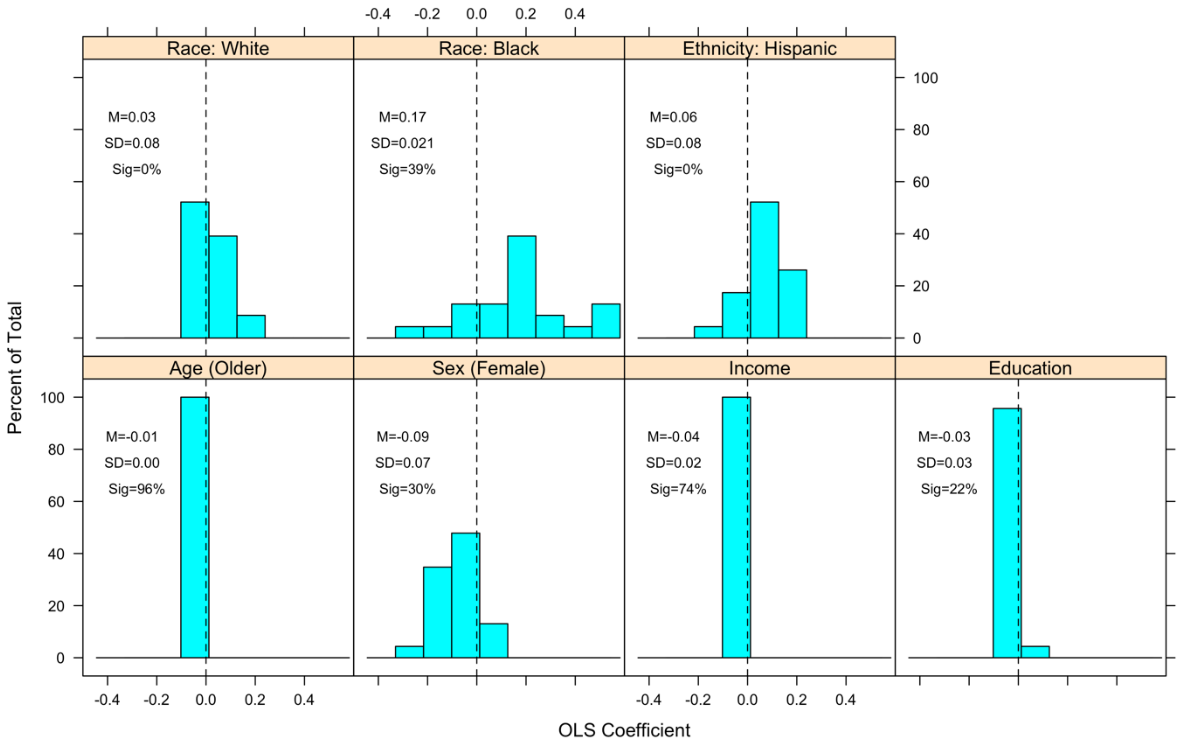

Figure SI 2: Distribution of odd ratios (based on logit coefficients) for dichotomous conspiracy belief questions, by sociodemographic characteristic. Mean, standard deviation, and percentage of cases where coefficient was statistically significant (*p*<0.05) appears in text. *P*-values corrected for multiple comparisons via Benjamini-Hochberg procedure.

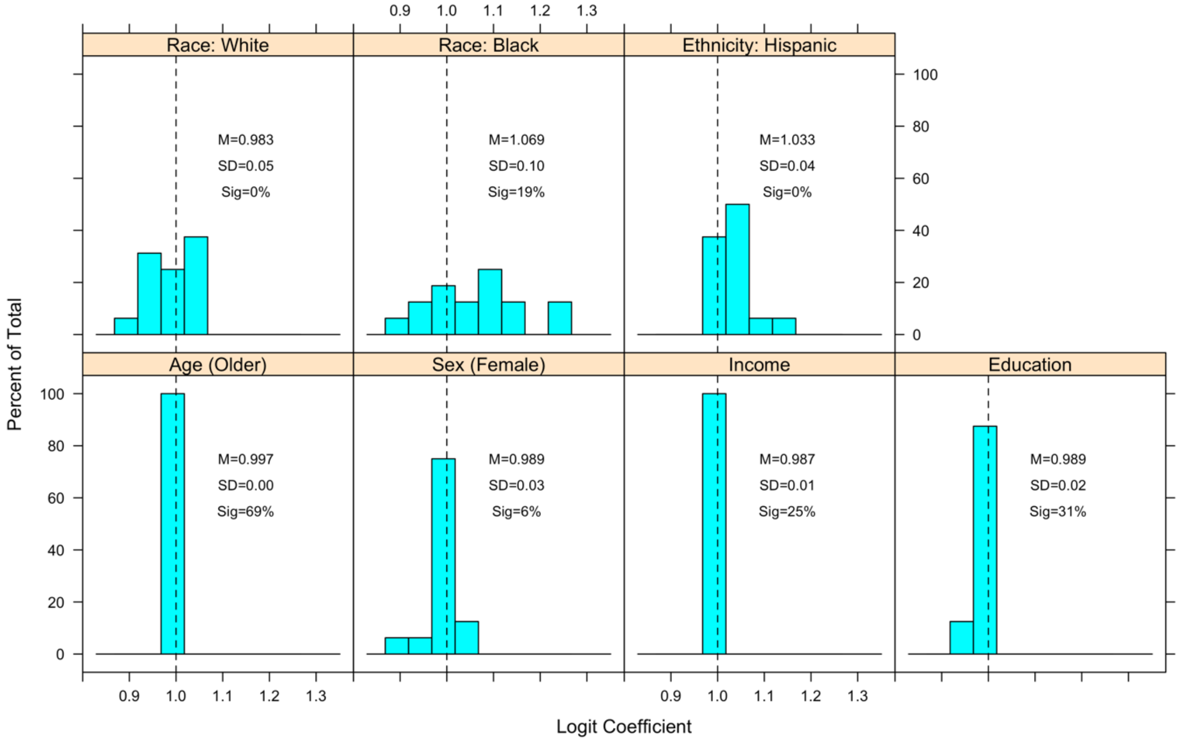

VI. Question wording for Study 2

The following were all answered using four-point scales ranging from "definitely" not true (1) to "definitely" true (4), with options of "probably" not true (2) and "probably" true (3) in the middle:

1. The US Government knowingly helped to make the 9/11 terrorist attacks happen in America on 11 September, 2001.
2. The truth about the harmful effects of vaccines is being deliberately hidden from the public.
3. Regardless of who is officially in charge of governments and other organisations, there is a single group of people who secretly control events and rule the world together.
4. The idea of man-made global warming is a hoax that was invented to deceive people.
5. Humans have made contact with aliens and this fact has been deliberately hidden from the public.
6. The AIDS virus was created and spread around the world on purpose by a secret group or organization.
7. The official account of the Nazi Holocaust is a lie and the number of Jews killed by the Nazis during World War II has been exaggerated on purpose. (The Holocaust denial question was not asked in Germany.)
8. The 1969 moon landings were faked.
9. Coronavirus is a myth created by some powerful forces, and the virus does not really exist.
10. The fatality rate of Coronavirus has been deliberately and greatly exaggerated.
11. The world's largest pharmaceutical companies are deliberately delaying or hiding the development of a vaccine that could end the Coronavirus pandemic, in order to drive up the price of the vaccine.

NOTE: The question about the Holocaust was not asked in Germany, as it is illegal to do so.

VII. Average belief by country for Study 2

Figure SI 3: Average conspiracy theory belief, by country. Horizontal bars represent 95% confidence intervals.

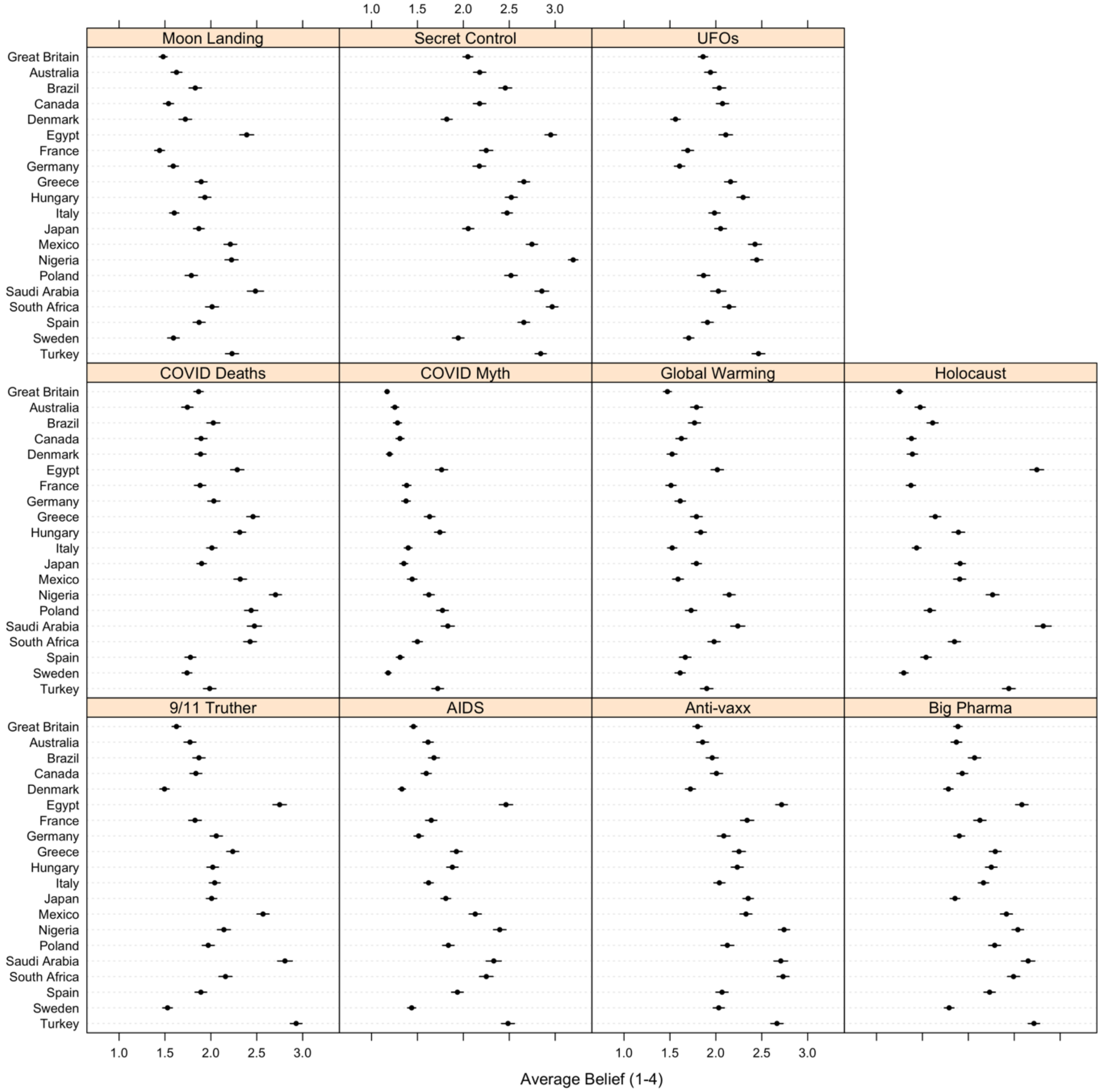

VIII. Complete correlations for Study 2

Figure SI 4: Correlations between **age (older)** and each conspiracy theory, by country. Horizontal bars represent 95% confidence intervals.

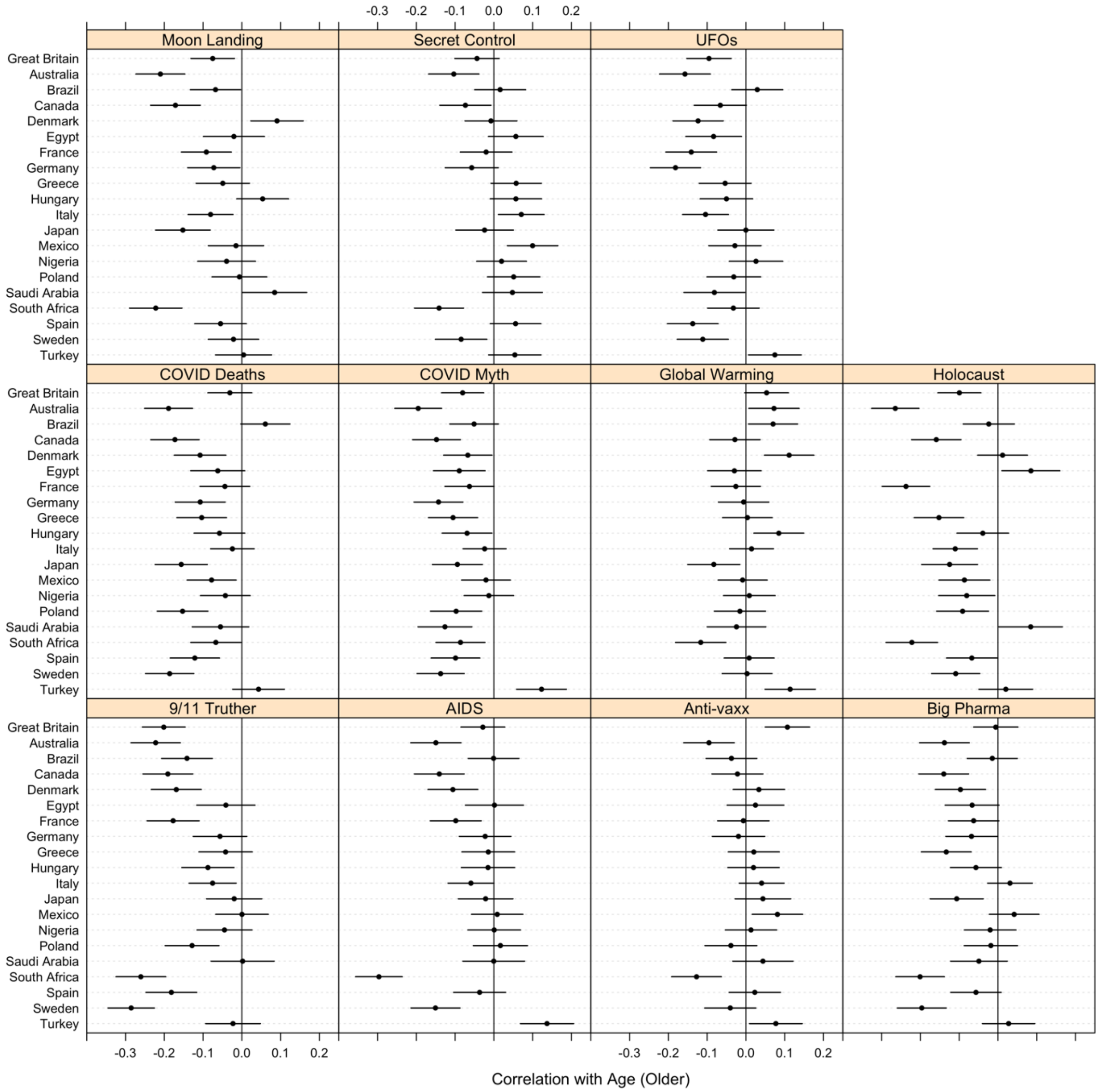

Figure SI 5: Correlations between **educational attainment** and each conspiracy theory, by country. Horizontal bars represent 95% confidence intervals.

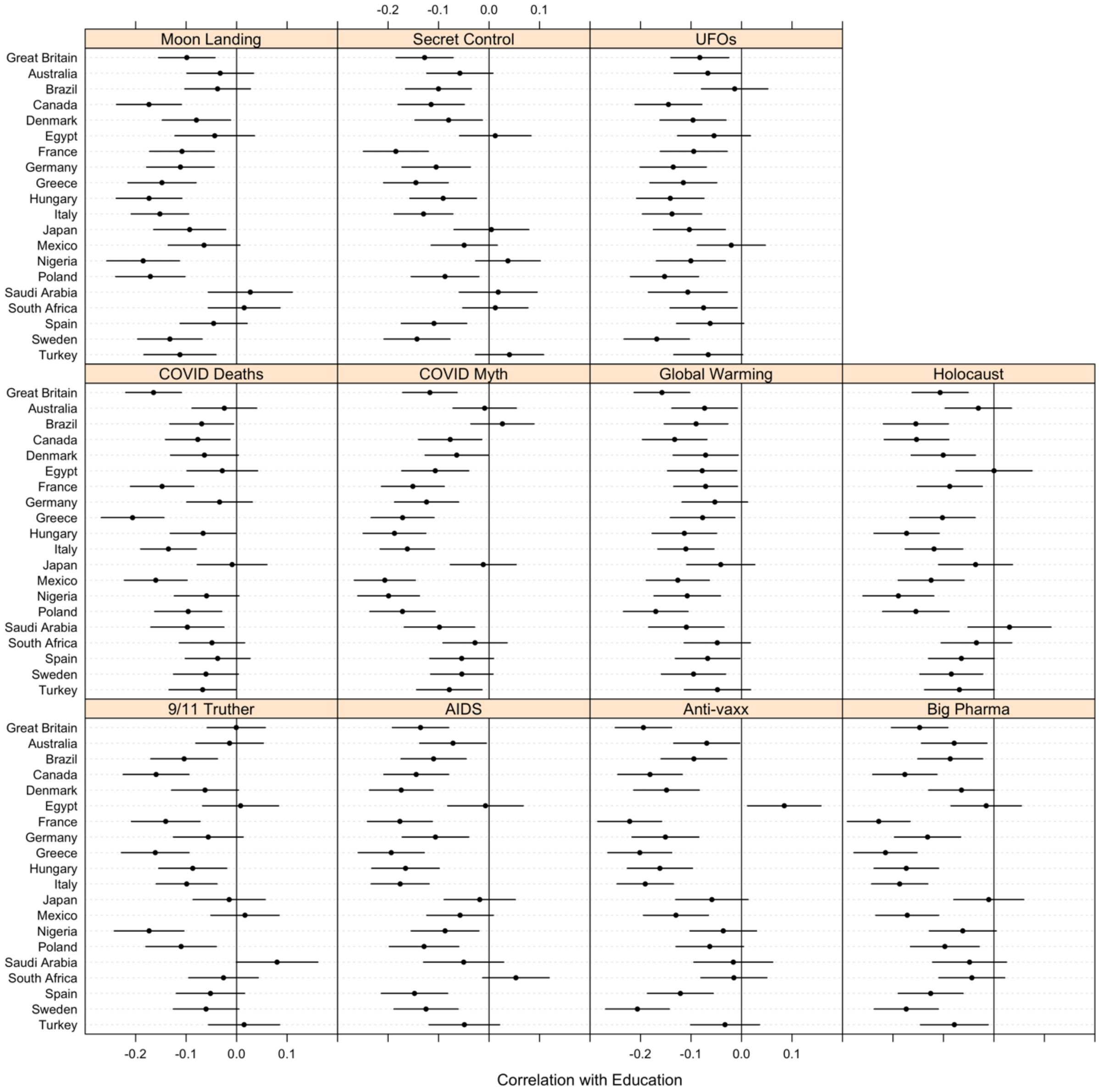

Figure SI 6: Correlations between **sex (female)** and each conspiracy theory, by country. Horizontal bars represent 95% confidence intervals.

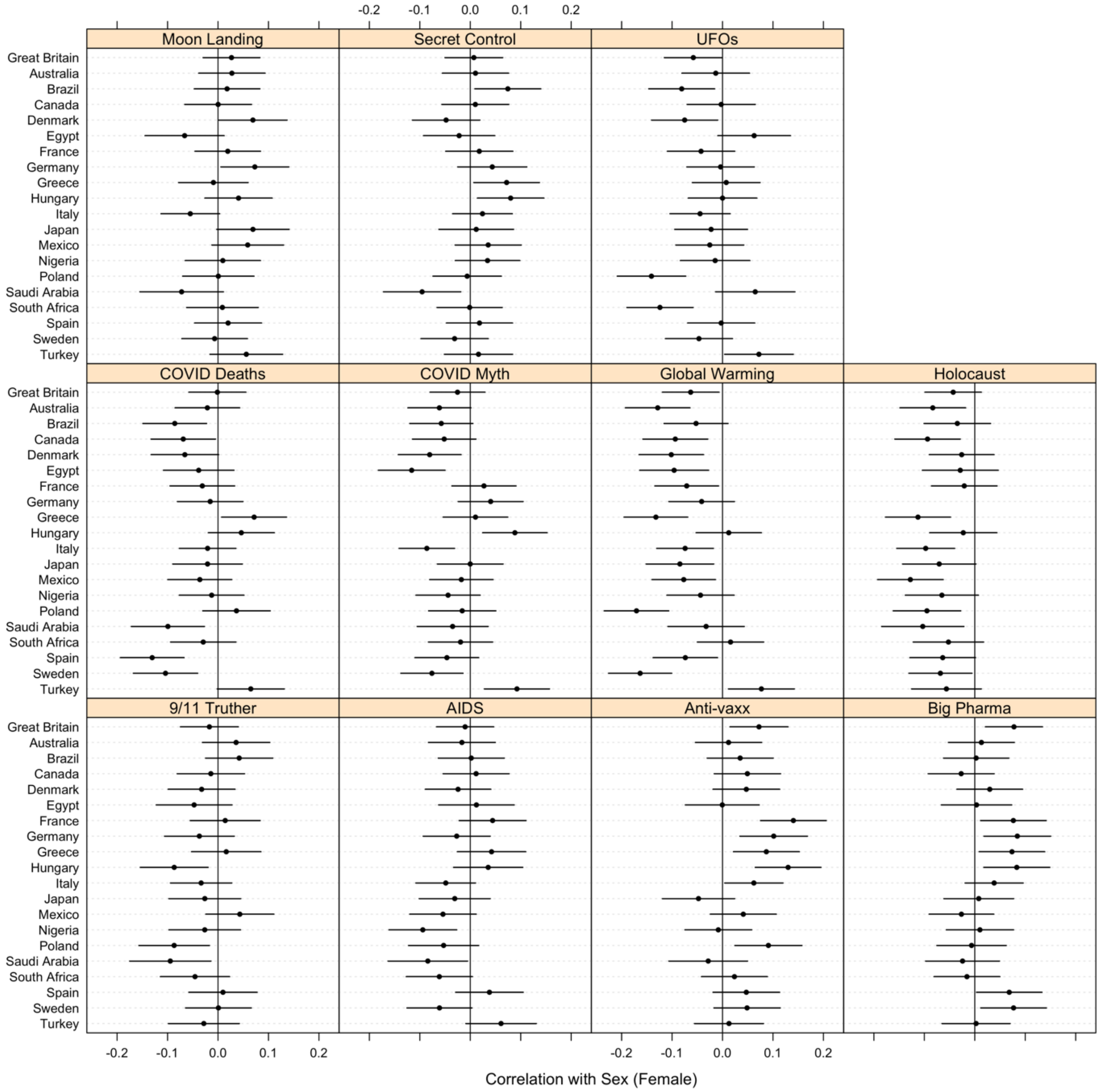

IX. Sociodemographic composition of samples for Study 4

Table SI 6: Sociodemographic information about all samples.

Characteristic	June 2022	May 2021	October 2020	June 2020	March 2020	July 2019	October 2018	October 2016	October 2012
Age	44	48	43	46.5	39	50	48	48	47
High school degree	97	97	97	98	95	99	91	89	89
Some college +	72	71	76	76	60	60	63	59	59
Female	55	50	51	51	52	52	52	52	52
White	78	67	68	60	65	62	70	73	73
Black	16	14	14	17	15	14	13	12	12
Hispanic	16	16	17	27	18	16	9	6	8
n	2,001	2,021	2,015	1,040	2,023	2,000	1,000	1,000	1,230

Note: All entries are percentages except age, which is the median.

Table SI 7: Details about samples from Study 4.

Polling Organization	Date Fielded	Sample Size	Sample/Sampling Procedure	University of Miami IRB Approval Date and Number
1. CCES	October 2012	1,230	Opt-in YouGov panelists; weighted to be representative	09/24/2012; 20120757
2. CCES	October 2016	1,000	Opt-in YouGov panelists; weighted to be representative	09/13/2016; 20120757 (MOD00013692)
3. CCES	October 2018	1,000	Opt-in YouGov panelists; weighted to be representative	07/07/2018; 20120757 (MOD00023764)
4. Qualtrics	July 2019	2,000	Quota sample; stratified to be representative	07/07/2019; 20190623
5. Qualtrics	March 2020	2,023	Quota sample; stratified to be representative	03/15/2020; 202000095
6. Qualtrics	June 2020	1,040	Quota sample; stratified to be representative	06/03/2020; 20200673
7. Qualtrics	October 2020	2,015	Quota sample; stratified to be representative	10/07/2020; 20201154
8. Qualtrics	May 2021	2,021	Quota sample; stratified to be representative	03/15/2021; 20210244
9. Qualtrics	June 2022	2,001	Quota sample; stratified to be representative	05/13/2022; 20220472

Note: “CCES”=Cooperative Congressional Election Study

References

1 Berinsky, A., Margolis, M. F., Sances, M. W. & Warshaw, C. Using screeners to measure respondent attention on self-administered surveys: Which items and how many? *Political Science Research and Methods* **9**, 430-437, doi:10.1017/psrm.2019.53 (2021).
